# The Importance of Hearing Screening and Central Auditory Processing in School-Aged Children

**DOI:** 10.3390/children11121450

**Published:** 2024-11-27

**Authors:** Piotr Henryk Skarżyński, Natalia Czajka, Ewelina Bukato, Rita Zdanowicz, Aleksandra Kołodziejak, Henryk Skarżyński

**Affiliations:** 1 Department of Teleaudiology and Screening, World Hearing Center, Institute of Physiology and Pathology of Hearing, 02-042 Warsaw, Poland; n.czajka@ifps.org.pl (N.C.); e.bukato@ifps.org.pl (E.B.); r.zdanowicz@ifps.org.pl (R.Z.); a.kolodziejak@ifps.org.pl (A.K.); 2 Institute of Sensory Organs, 05-830 Kajetany, Poland; 3 Oto-Rhino-Laryngology Surgery Clinic, World Hearing Center, Institute of Physiology and Pathology of Hearing, 02-042 Warsaw, Poland; h.skarzynski@ifps.org.pl

**Keywords:** hearing screening, pure tone audiometry, central auditory processing disorder

## Abstract

Background/Objectives: The aim of the study was to evaluate the prevalence of undiagnosed hearing impairment or central auditory processing disorders in children from I and VIII grades of primary schools in Warsaw. Methods: The participants in the study were 15,659 pupils from classes I and VIII attending primary schools in Warsaw. As part of the study, the hearing threshold for air conduction at frequencies of 0.5–8 kHz was determined, and a test assessing central auditory processing was performed: FPT (Frequency Pattern Test), DDT (Dichotic Digit Test), the Auditory Behaviour Scale (SAB), and an interview questionnaire developed for the program. Results: Abnormal test results were found in 1946 children, of which abnormal hearing screening test results were found in 678 children tested, while reduced central auditory processing results were found in 1268 children. Conclusions: The program implemented draws attention to the fact that tests of central auditory processing are included in the testing protocol. As has been shown, peripheral hearing testing alone is not sufficient to exclude abnormalities concerning the sense of hearing.

## 1. Introduction

The sense of hearing allows a multifaceted interpretation of the sounds we hear. It gives us the possibility to read the auditory reality in which humans function. However, one of its most important tasks is to enable communication through verbal speech. Hearing deprivation at every stage of life has a number of negative consequences; hearing problems can appear in a person at any age, either suddenly or gradually, remaining in many cases unnoticed [1].

Hearing disorders especially in children at the speech development stage require prompt diagnosis and treatment. Auditory deprivation resulting in a lack of sensory stimulation in the auditory cortex area causes a number of molecular degenerative changes in the nerve cells. This has the effect of stunting the functional development of the auditory cortex, the absence or difficulty of speech development, and, in cases of total deafness, the inability of the child to develop verbal communication [2]. Auditory deprivation has a significant impact on a child’s development in all aspects of life:

1. Speech and language development: Hearing plays a key role in speech acquisition and language learning. Children who experience auditory deprivation may have difficulties in the proper development of speech and, subsequently, language, i.e., acquiring vocabulary and developing communication skills.
2. Education and school skills: Auditory deprivation can affect a child’s learning abilities and ability to learn at school. Good listening comprehension, communication with teachers and peers, and participation in lessons themselves may be impaired. Hearing impairment can also translate into school skills such as reading and writing.
3. Social functioning: Hearing is the sense on which much of the construction of social interaction is based. It plays a key role in establishing relationships with peers. Hearing deprivation can consequently lead to relationship-building problems and even social isolation.
4. Spatial orientation: hearing disorders, particularly unilateral, can affect a child’s spatial orientation, especially in the localization of sounds.
5. Emotional functioning: Recognition and expression of emotions are to some extent based on auditory experience. The voice is a carrier of emotions and its interpretation helps in interpersonal relationships. Children with difficulties in the auditory area may have difficulties in understanding and expressing emotions. Overlapping difficulties in social relationships can also cause the child to close off from close relationships, and feelings of security can also be disrupted.
6. Cognitive functioning: hearing and sound stimulation play a very important role in the child’s cognitive development, influencing and shaping thought processes, memory, and concentration [3,4].

The hearing organ, like the other senses, is divided into a peripheral part that collects sensory information from the environment and a central part that processes this information. Proper hearing, therefore, depends on the proper function of all parts of the auditory pathway, both those that conduct sound waves (the external and middle ear) and those that transform signals into bioelectrical impulses (the inner ear), which are transmitted through the auditory nerve to the central nervous system. If abnormalities occur in any of the components of the auditory pathway, hearing impairment can occur.

The normal conduction and perception of sounds by the peripheral hearing organ and their processing at the level of the central nervous system are interdependent processes. Typical symptoms indicating the possibility of hearing disorders include, among others, the following:

- Delayed speech development or limited language skills, late talkers;
- slurred speech,
- ‘Losing’ word beginnings or word endings;
- Support of auditory communication by lip reading;
- Problems with auditory writing (the child writes as he/she hears);
- Delayed or no answer when called (even by name);
- Difficulty understanding instructions given in a noisy environment;
- Difficulty understanding complex instructions;
- Frequent requests to repeat, questioning, difficulty understanding a question;
- Making the TV, radio, and music players louder;
- Difficulties in localizing the source of a sound;
- Talking loudly in a quiet environment;
- Hypersensitivity (to loud noises);
- Hearing sounds that others cannot hear (humming, squeaking, whistling, ringing) [5].

Referring to the above difficulties related to hearing disorders, it is worth mentioning that central auditory processing disorder is sometimes confused with hearing impairment, although peripheral hearing is normal. As defined by the American Speech-Language-Hearing Association (ASHA), these are difficulties in processing the auditory stimuli in the central part of the nervous system [6].

Hearing screenings are designed to detect hearing problems. They make it possible to quickly identify disorders in the area of this sense, which is crucial for effective treatment or rehabilitation and to prevent further aggravation.

The years 1992–1994 can be considered the beginning of hearing screening in Poland, when, thanks to the cooperation of docent Maria Góralówna and the Diagnostic, Treatment and Rehabilitation Centre ‘Cochlear Center’, managed by professor Henryk Skarżyński, hearing screening was carried out in newborns and infants from the risk group. In 1998, in Milan, at a joint meeting of specialists from all over the world, the European Consensus on Universal Hearing Screening in Newborns was signed, of which the Polish signatory was Professor Henryk Skarżyński [7]. Severe hearing impairment occurs in approximately 2–4 per 1000 newborns [8]. Sometimes, hearing loss can progress or become apparent within the first few years of life. Some children may also lose their hearing later as a result of experiencing infections, infectious diseases, meningitis, head trauma, etc. The results of the Institute of Physiology and Pathology of Hearing’s hearing screening programs for school-aged children show that up to one in five children may have hearing problems [9,10,11,12,13].

In Poland, only newborns are covered by universal hearing tests. Various hearing screening programs are carried out in different age groups as part of various prevention campaigns. One of these is the program of the Office of Health Policy of the City of Warsaw, which has been running since 2007. The Institute of Physiology and Pathology of Hearing has been implementing the hearing screening program among students of Warsaw schools since the beginning of the program. In the 2023/2024 school year, the tests covered children from grades I and VIII and the diagnosis included hearing screening and, for the first time as part of this program, central auditory processing.

The aim of the study was to evaluate the prevalence of undiagnosed hearing impairment or/and central auditory processing disorders in children from I and VIII grades of primary schools in Warsaw.

## 2. Materials and Methods

**Figure 1:**
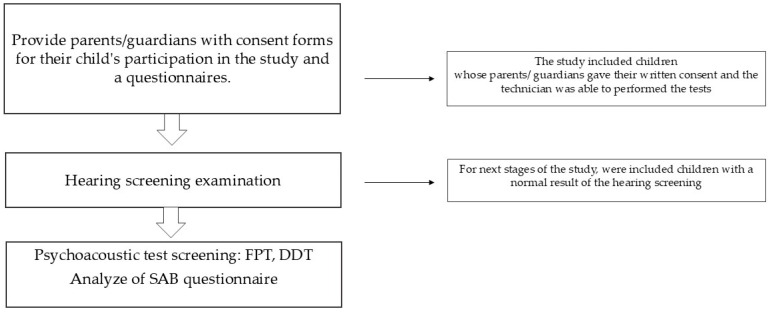
Scheme of the test procedure.

The “Health Policy Programme for Early Detection of Hearing Disorders among Pupils in Classes I and VIII of Elementary Schools in the Capital City of Warsaw for 2023–2025” involved pupils starting and finishing primary schools in Warsaw in the 2023–2024 school year. Inclusion criteria included (1) children attending the first or eighth grade of primary school; (2) children whose parents gave written consent to participate in the study; and (3) children who had no previously diagnosed hearing problems. Exclusion criteria included (1) children with diagnosed hearing loss and (2) children in whom, due to lack of cooperation, the hearing screening and central auditory processing disorder screening could not be carried out. A scheme of the test procedure (Figure 1) is shown in the figure below.

The screening program used the Sensory Examination Platform^®^ (https://medincus.pl/oferta/uslugi/platforma-badan-zmyslow/?temat=o-platformie-badan-zmyslow, accessed on 27 April 2024). This device was developed by the Institute of Hearing Physiology and Pathology in Kajetany, Poland in cooperation with the Institute of Sensory Organs Kajetany, Poland. The Sensory Examination Platform^®^ is a portable device with which screening tests of the senses, including hearing and speech, can be carried out quickly and easily. It is equipped with calibrated Sennheiser HDA 200 audiometric headphones and a button (Senses Examination Platform is manufacturer by Center of Hearing and Speech MEDINCUS Mokra 7 St., 05-830 Kajetany, Poland). Using the Internet, the PBZ can connect to the Central System, into which the test results are collected and evaluated [14].

### 2.1. Study Procedure

Hearing screening and processes assessing auditory processing were carried out at primary schools in the city of Warsaw using the Sensory Examination Platform^®^. All tests were selected so that they can be performed on the Sensory Examination Platform^®^ and, in line with the idea of screening, as quickly as possible. Each test was performed by an experienced technician from the Institute of Physiology and Pathology of Hearing in a specially prepared quiet room in the school. Every child whose parents gave written consent for the screening was tested. Parents also completed a questionnaire. Filling in the audiological questionnaire by parents/guardians was not necessary for the child to participate in the study.

### 2.2. Hearing Screening Test

The air-conduction screening pure tone audiometry was performed binaurally in the frequency range from 500 Hz to 8000 Hz, omitting the semi-octaves 3000 Hz and 6000 Hz unless necessary. An abnormal result was considered when the hearing threshold value for air conduction was greater than 20 dB HL at any frequency in either the left or right ear and the result was classified as abnormal by the doctor [11].

### 2.3. FPT (Frequency Pattern Test)

One of the tests that assessed auditory processing was the FPT. During this test, the child heard three sounds at different low (880 Hz) and high (1122 Hz) frequencies [15]. The child’s task is to repeat sequences of these sounds as he or she hears them, e.g., low-low-high. The screening test was completed after 20 samples [16].

### 2.4. DDT (Dichotic Digit Test)

The second test to assess auditory processing was the DDT. During this test, the child heard two different pairs of digits (numbers from 1 to 10) in the right ear and the left ear at the same time. The pupil’s task was to repeat as many of the digits he or she heard as possible. The test ended after the children had completed their ten attempts [17,18,19].

### 2.5. Audiological Questionnaire

The questionnaire was developed by the Institute of Physiology and Pathology of Hearing team for the screening program. It contains 11 questions concerning, among other things, the parent’s subjective assessment of the child’s hearing status, speech development, tinnitus, and the care of specialists.

### 2.6. Scale of Auditory Behaviors (SAB)

The Scale of Auditory Behaviors consists of 12 statements relating to a child’s hearing difficulties. Scores are considered from 1, meaning very often, to 5, meaning never. A higher score suggests less difficulty with possible auditory processing. A score below 35 was considered abnormal [20,21].

All children with abnormal results of hearing and auditory processing disorder analyzed by the doctor were referred for a follow-up visit. Such a visit could take place at the Institute of Physiology and Pathology of Hearing following a referral from a pediatrician. In some children, after diagnostics performed by a specialist in the APD field, auditory processing disorder therapy was suggested.

## 3. Results

A total of 21,599 families were asked to participate in the study. The study included 15,659 children whose parents/guardians gave their written consent. Pupils in the first grades made up the vast majority of this edition of the program and numbered 10,743, or 69% of all children. Eighth graders were less numerous and accounted for 31% of all eligible children, or 4916 pupils. The gender split in the study population was very similar. First-grade girls accounted for 5327—49.6%, while first-grade boys accounted for 5415—50.4%. In Class VIII, the gender split was also similar, with girls accounting for 2419—49.2%—and boys 2497—50.8%. The mean age of children attending for the first grade was 6.7 years. The mean age of children attending for the eighth grade was 13.2 years. All data are shown in Table 1.

After synchronization (online transfer of the collected data by a technician to the main computer system of screening located in the Institute of Physiology and Pathology of Hearing) of the results (hearing screening, FPT and DDT tests, an audiological questionnaire, and Scale of Auditory Behaviors) to the Central System, the data that were automatically qualified as abnormal by the system were additionally analyzed by a specialist. The system has the abnormal result (concerning hearing screening) setting coded as greater than or equal to 25 dB at least one frequency. The doctor assessed them on the basis of the pure tone audiometry screening. The results for auditory processing disorders were analyzed on the basis of the SAB questionnaire and also according to reference values for FPT, and DDT for children [17]. Scores in the SAB questionnaire that were below 35 were classified as requiring follow-up [21].

The hearing screening test was performed on 15,659 children. Of the 15,659 pupils classified for hearing screening, a total of 1946 children—12.4%—had an abnormal score in hearing screening and auditory processing disorder as well. Analysis of the results of the hearing screening showed abnormal results (greater than or equal to 25 dB at least one frequency) in 678 pupils; this was 34.8% of the pupils with an abnormal result. Moreover, 1268—65.2%—of children had an abnormal result in auditory processing. Finally, the auditory processing disorder tests included 14,981 pupils with a normal hearing screening test. Moreover, 23 children did not participate in the study due to lack of cooperation with technicians and were not included in any calculations as well.

Furthermore, of the 206 people who attended the follow-up visit, the need to implement central auditory processing therapy was obtained in as many as 134—65%. In addition, 31—15%—of the individuals should present for verification of qualification for therapy approximately 12 months after first diagnosis. All data are shown in Table 2.

## 4. Discussion

In Poland, screening programs for hearing are common only for newborns. These programs are effective, although it is not possible to verify hearing in the later years of a child’s life through general screening. Newborn screening is a very important aspect of child screening, but it cannot ensure that hearing status will not change with age, especially in the first decade of a child’s life [22]. Follow-up of such testing for older children is possible through various programs mandated by, for example, municipalities [11,23]. The screening program is also performed in other parts of the country. The authors used a hearing screening test performed on Sensory Examination Platform^®^ as in this publication [13,24]. The authors emphasize the important value of screening among children. Detection of hearing loss provides an opportunity for early implementation of a full diagnosis and appropriate treatment.

In our study, prevalence of hearing loss was observed in 4.3% of all children qualified for hearing tests, and it was similar in boys and girls. Skarzynski et al. [24], in their publication, declare that the number of abnormal results in hearing screening is as high as 9.4%; however, the number of examined population was more numerous than in the present study and the examinations were performed in rural areas; hence, the difference in prevalence may be due differing socioeconomic status in these two populations. It should be noted that when it comes to identifying abnormal hearing in younger children (in first grade) compared to older ones (eighth grade), we have a great difference. The difference may be due to (1) a larger group of first-grade students participating in the study and/or (2) ear infections, e.g., otitis media, especially concerning young children [25,26]. The main aim of screening is to identify a group of people who may have hearing problems [13,24,27]. Moreover, performing the hearing screening in two steps, i.e., first performing the hearing test, then testing for auditory processing, eliminates children who have a hearing disorder first. This allows for earlier separation of pupils who may be misclassified as having central nervous system auditory processing problems. This is why we chose not to include pupils with hearing loss in our auditory processing examination. The 2023–2024 edition of the screening program focuses not only on performing a hearing screening test, but also includes tests to assess auditory processing. Previously, tests assessing auditory processing were carried out in Warsaw primary schools between 2008 and 2010, at which time only the DDT test was performed and the percentage of children diagnosed with auditory processing disorder would be 11.4% of tested children for 7-year-old and 11.3% of tested children for 12-years-olds [12]. Currently, in addition to the DDT test, the FPT test has also been performed, which gives more insight into possible auditory processing problems in children. In our study, the prevalence of auditory processing disorder is 8.1%. Amizadeh et al. [28] showed the prevalence of APD was 8.03%, and this result is very similar to our results. However, it should be noted that the number of students surveyed was much smaller. Research conducted by Shreyas [29] and Nagao [30] showed the prevalence at the level of, respectively, 0.7% and 0.2%. The reasons for such differences could be the difference in the number of people surveyed, the socioeconomic status of families, or different areas of residence. There are many equal screening methods for auditory processing [31]; among others, use of questionnaires such as the Scale of Auditory Behaviors, which showed a high correlation with behavioral tests and can be used as a screening tool for assessing auditory processing [20,32]. Adding to hearing screening tests that assess auditory processing has made it possible to identify more children who may have problems in everyday functioning. By definition, these are children who have normal hearing but function as hearing-impaired individuals and have great difficulty understanding speech in noisy environments [33,34,35,36,37]. The implementation of tests to assess auditory processing will be particularly important in children beginning primary school. At this stage of their lives, they experience difficulties with speech in noisy surroundings and with concentrating on various environmental stimuli [20,38,39,40].

Early diagnosis of possible hearing problems is an important factor in a child’s psychosocial development, and promptly implemented rehabilitation or surgical intervention will minimize the risk of developmental delay [35,41,42].

## 5. Conclusions

Verification of peripheral hearing is the basis of hearing screening. However, it may not be sufficient. Despite normal hearing, the child may function as a hearing-impaired person due to dysfunction in the processing of auditory stimuli. The need to implement additional testing, i.e., tests to assess central auditory processing, will be key to a comprehensive check of the child’s auditory functioning.

## Figures and Tables

**Table 1:** Numbers of pupils included in the study.

Numbers of Pupils Total			
n = 15,659			
Numbers of pupils in first grade		Numbers of pupils in eighth grade	
n = 10,743 (69%)		n = 4916 (31%)	
M = 6.7 years		M = 13.2 years	
Numbers of girls	Numbers of boys	Numbers of girls	Numbers of boys
n = 5327 (49.6%)	n = 5416 (50.4%)	n = 2419 (49.2%)	n = 2497 (50.8%)

n—number of pupils; M—mean age of pupils.

**Table 2:** Abnormal results for hearing screening and auditory processing.

Total Number of Pupils with Abnormal Results			
1946 (12.4%)			
Number of pupils with abnormal results after hearing screening		Number of pupils with abnormal results in auditory processing	
678 (34.8%)		1268 (65.2%)	
Number of pupils from first grade with abnormal results after hearing screening	Number of pupils from eighth grade with abnormal results after hearing screening	Number of pupils from first grade with abnormal results in auditory processing	Number of pupils from eighth grade with abnormal results in auditory processing
n = 586 (3.7%)	n = 92 (0.6%)	n = 795 (5.3%)	n = 473 (3.15%)
n = 281 (girls)	n = 42 (girls)	n = 284 (girls)	n = 197 (girls)
n = 305 (boys)	n = 50 (boys)	n = 511 (boys)	n = 276 (boys)

n—number of pupils.

## Data Availability

The raw data supporting the conclusions of this article will be made available by the authors upon request. The data are not publicly available due to patients privacy.
